# Effects of Voluntary Wheel-Running Types on Hippocampal Neurogenesis and Spatial Cognition in Middle-Aged Mice

**DOI:** 10.3389/fncel.2018.00177

**Published:** 2018-06-26

**Authors:** Yi-Qing Huang, Cheng Wu, Xiao-Fei He, Dan Wu, Xia He, Feng-Yin Liang, Guang-Yan Dai, Zhong Pei, Guang-Qing Xu, Yue Lan

**Affiliations:** ^1^Department of Rehabilitation Medicine, The First Affiliated Hospital, Sun Yat-sen University, Guangzhou, China; ^2^Department of Rehabilitation Medicine, Guangzhou First People’s Hospital, Guangzhou Medical University, Guangzhou, China; ^3^Department of Neurology, National Key Clinical Department and Key Discipline of Neurology, Guangdong Key Laboratory for Diagnosis and Treatment of Major Neurological Diseases, The First Affiliated Hospital, Sun Yat-sen University, Guangzhou, China; ^4^Department of Integrated Traditional and Western Medicine, Sichuan Bayi Rehabilitation Center, Chengdu, China; ^5^Department of Rehabilitation Medicine, Beijing Tiantan Hospital, Capital Medical University, Beijing, China

**Keywords:** wheel running, spatial memory, neurogenesis, aging, hippocampus

## Abstract

While increasing evidence demonstrated that voluntary wheel running promotes cognitive function, little is known on how different types of voluntary wheel running affect cognitive function in elderly populations. We investigated the effects of various voluntary wheel-running types on adult hippocampal neurogenesis and spatial cognition in middle-aged mice. Male C57BL6 and Thy1-green fluorescent protein (GFP) transgenic mice (13 months) were equally assigned to one of the following groups: (1) T1: no voluntary wheel running; (2) T2: intermittent voluntary wheel running; and (3) T3: continuous voluntary wheel running. The Thy1-GFP transgenic mice were used to specifically label granule cells, since Thy-1 is a promoter for neuronal expression. Behavioral evaluations suggested that intermittent voluntary wheel running improved Morris water maze performance in middle-aged mice. The number of BrdU-positive cells was significantly higher in both intermittent and continuous voluntary wheel running compared with no voluntary wheel running. However, only intermittent voluntary wheel running facilitated the newborn cells to differentiate into granule cells, while newborn cells tended to differentiate into astrocytes and repopulation of microglia was also enhanced in the continuous voluntary wheel-running group. These results indicated that intermittent voluntary exercise may be more beneficial for enhancing spatial memory. Effective improvement of hippocampal neurogenesis was also caused by intermittent voluntary wheel running in middle-aged mice.

## Introduction

Increased human longevity has magnified the negative impact that aging can have on cognitive function (Foster et al., 2017), especially on hippocampal-dependent functions (Hullinger and Puglielli, 2017), such as spatial learning and episodic memory (Foster, 2006). Importantly, the part which is most vulnerable to neurodegenerative disease pathology is the same part that is susceptible to synaptic loss during aging in the nervous system, including the hippocampus, which directly contributes to age-related cognitive impairment (Canas et al., 2009). An imaging study demonstrated hippocampal atrophy in elderly people (Small et al., 2002). In addition, hippocampal neurogenesis decreases with aging (Lichtenwalner et al., 2001).

There is substantial evidence from experimental studies indicating that physical exercise is a key factor in promoting cognitive function and overall brain health, particularly in elderly populations (Cotman et al., 2007; Hillman et al., 2008). Several physical training approaches in cognitive rehabilitation have been developed, such as voluntary and forced training. However, very little is known about the different contribution of different physical training approaches to the cognitive function in aging. As to forced training, comparing different types by controlling exercise intensities showed that low-intensity forced training seemed more effective in enhancing hippocampal neurogenesis and improving cognitive function than high-intensity forced training (Shen et al., 2013; Shih et al., 2013; Shimada et al., 2013; Inoue et al., 2015). However, the effects of voluntary exercise types on hippocampal neurogenesis and cognitive function in aging remain unclear. Interestingly, a previous research reported that the cessation of voluntary wheel running (housed in a running wheel cage for 8 weeks and subsequently in a standard cage for 8 weeks) increased anxiety-like behavior and impaired adult hippocampal neurogenesis in mice (Nishijima et al., 2013). Since high intensity interval training (HIIT) has been prevalent recent years, we speculated whether voluntary wheel running at intervals would eliminate the negative impact of the cessation and even be more beneficial than continuous exercise. Thus, in the current study, we compared the effects of various types of voluntary wheel running, including no voluntary wheel running, intermittent voluntary wheel running and continuous voluntary wheel running.

In an attempt to improve our understanding of the impact of different types of voluntary exercise on brain function, we investigated the effects of different voluntary wheel-running protocols on spatial memory performance in middle-aged mice. Furthermore, we investigated hippocampal neurogenesis and gliogenesis responding to different voluntary wheel-running types, which might interpret different performance in the cognitive task. We hypothesized that intermittent voluntary wheel running may be more beneficial for enhancing spatial memory due to effective improvement of hippocampal neurogenesis.

## Materials and Methods

### Animals and Ethics Statement

Eighteen adult male C57BL6 and 18 adult male Thy1-green fluorescent protein (GFP) transgenic mice (Model Animal Research Center of Nanjing university, Stock Number: 003782) at 13 months of age were used. All animals were housed with food and water provided *ad libitum* under a 12:12 light-dark cycle (lights on from 7:00 to 19:00) with controlled temperature (20–26°C) and humidity. This study was carried out in accordance with the recommendations of the Animal Research Committee of the First Affiliated Hospital of Sun Yat-sen University. The protocol was approved by the Animal Research Committee of the First Affiliated Hospital of Sun Yat-sen University. All efforts were made to minimize the suffering and number of animals used in this study.

### Voluntary Wheel Running

To the best of our knowledge, there have been no exercise protocols designed to investigate different voluntary running types. Such being the case, we designed our own exercise protocols, combining the protocols reported by Nishijima et al. (2013) and the mode of HIIT. From a conceptual viewpoint, intermittent voluntary wheel running could be defined as repeated short-term voluntary wheel running at regular intervals of cessation. Continuous wheel running could be defined as long-term voluntary wheel running without cessation. The main difference between them is the duration of voluntary wheel running.

**Figure 1:**
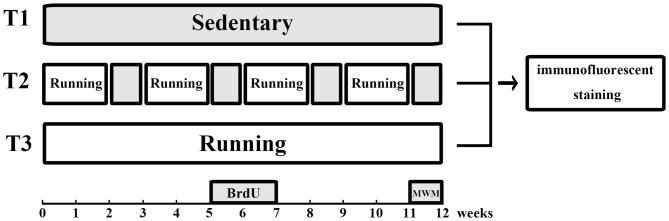
Experimental design. Animals in the T1 (no voluntary wheel running) group were housed in standard laboratory cages without access to running wheels. Mice in the T2 (intermittent voluntary wheel running) group were housed with free access to running wheels for 2 weeks and without access to running wheels for the next week. This procedure was repeated for a period of 12 weeks. Mice in the T3 (continuous voluntary wheel running) group were housed with free access to running wheels for 12 weeks. All mice were injected intraperitoneally with BrdU during week 6 and week 7. The water maze task was performed during the last week, followed by immunofluorescence staining. BrdU, 5-bromo-2′-deoxyuridine; MWM, Morris water maze.

Animals were randomly divided into three groups as shown in Figure 1, including T1: no voluntary wheel running, T2: intermittent voluntary wheel running, and T3: continuous voluntary wheel running; each group included six male C57BL6 and six male Thy1-GFP transgenic mice. Animals in the T1 group were housed in polypropylene cages (36 cm L × 20 cm W × 14 cm H) for 12 weeks. Mice in the T2 group were first housed in polypropylene cages of the same size, with a 16-cm-diameter running wheel for 2 weeks and subsequently housed in cages without running wheels for 1 week. This schedule (running wheel for 2 weeks, followed by no running wheel for 1 week) was followed for a period of 12 weeks. Mice in the T3 group were housed in polypropylene cages (36 cm L × 20 cm W × 14 cm H) with a 16-cm-diameter running wheel for 12 weeks. All mice were housed in groups (three animals per cage) and the groups of cage mates were not changed throughout the experiment (Luo et al., 2007), because social isolation is known to increase anxiety-like and depression-like behaviors (Koike et al., 2009) and to suppress exercise-induced neurogenesis in the hippocampus (Stranahan et al., 2006). Mice were deliberately not housed in cages with locked wheels because they would climb in locked wheels and we wanted to keep physical activity to a minimum in the T1 (sedentary) group (Rhodes et al., 2003; Clark et al., 2008). All mice were injected intraperitoneally with 5-bromo-2′-deoxyuridine (BrdU; 50 mg/kg, seven times at 48-h intervals, catalog number 59-14-3, Sigma, USA) during week 6 and week 7 (Zhao et al., 2003; Kee et al., 2007a).

### Morris Water Maze

The water maze procedure was performed during the 12th week according to the protocols of van Praag and Akers (van Praag et al., 1999; Akers et al., 2014). The maze consisted of a circular tub (120 cm in diameter, 50 cm in height) and a white circular platform (10 cm). The tub was surrounded by a curtain that was located about 1 m from the tub wall and painted with distinct geometric cues; the water (24 ± 1°C) was made opaque with white tempera paint to conceal the platform. Over five consecutive days, the platform was submerged 1 cm under the surface of water in the center of one of the pool quadrants. Mice received four trials (up to 60 s) per day from each of four different start locations. Animals that failed to locate the platform within the allotted 60 s were gently guided to the platform. All mice remained on the platform for 10 s at the end of each trial. On day 6, the platform was removed and a single 60-s probe trial was conducted. Swim paths were recorded by an overhead video camera and tracked by the automated software (San Diego Instruments, CA, USA), including latency to reach the platform during water maze training, swimming speed, number of times the mice crossed the target area (former platform), and time spent in each quadrant during the probe trial.

### Tissue Preparation and Immunochemistry

Two hours after the water maze task, a total of 36 mice (*n* = 12 per group) were deeply anesthetized with sodium pentobarbital and transcardially perfused with 50 ml of ice-cold saline, followed by 50 ml of 4% (w/v) paraformaldehyde (PFA) in phosphate-buffered saline (PBS; pH 7.4). Their brains were removed and incubated overnight in PFA and dehydrated in 20%–30% sucrose in PBS. Coronal sections of 10 μm were serially cut through the entire rostral caudal extent of the dentate gyrus (DG) using a microtome (Leica) at intervals of 10 in consecutive frozen sections and stored at −80°C for later immunofluorescence staining.

For immunofluorescence staining, all sections were pretreated for BrdU staining by denaturing DNA, and then sections were microwaved with citric acid buffer (pH 6.0) for 5 min. After cooling, the sections were treated with 0.3% Triton and 10% goat serum for 1 h at room temperature. Next, sections were incubated with primary antibody (1:400 anti-NeuN antibody, catalog number MAB377, Chemicon, USA; 1:500 anti-BrdU antibody, catalog number ab152095, Abcam, USA; 1:400 anti-glial fibrillary acidic protein (GFAP) antibody, catalog number G3893, Sigma, USA; 4′,6-diamidino-2-phenylindole (DAPI), catalog number F6057, Sigma, USA; 1:400 anti-ionized calcium-binding adapter molecule 1 (Iba-1) antibody, catalog number 019–19741, Wako, Japan) overnight at 4°C, followed by secondary antibodies (1:300 Alexa-Fluor-488-conjugated goat anti-mouse IgG2a antibody, Life Technologies, catalog number A21131; 1:300 Alexa-Fluor-555-conjugated goat anti-rabbit IgG1 antibody, Life Technologies, catalog number A31572) at room temperature in PBS containing 10% NGS for 1 h. Slices were mounted onto slides, embedded with SlowFade^®^ Gold (Invitrogen), and enclosed with a coverslip.

Immunofluorescence was observed under a confocal microscope (Leica, DM6000, German). The numbers of co-labeled BrdU-positive cells were counted throughout the subgranular and granular cell layers of the entire DG in both sides using Leica Application Suite X software. About 10 sections per sample were taken into account. ImageJ software (National Institutes of Health, Bethesda, MD, USA) was used to analyze the results of immunohistochemistry.

### Data and Statistical Analyses

All statistical analyses were conducted using SPSS 19.0 software (Armonk, NY, USA). Repeated-measures analyses of variance (ANOVA) was used for analyzing changes over time. One-way ANOVA was used for simple group comparisons. LSD-*t* test was used for *post hoc* comparisons. Pearson’s correlation was used for correlation analysis. A *P*-value < 0.05 was considering statistically significant. Data are expressed as the mean and standard error of the mean (SEM).

## Results

### Performance in the Morris Water Maze Task

**Figure 2:**
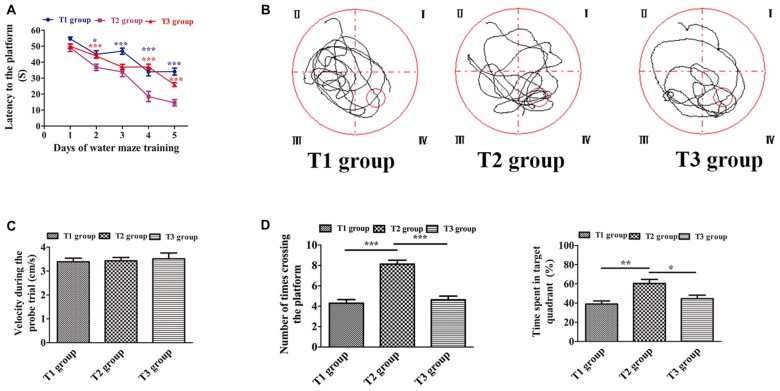
Intermittent voluntary wheel running improved water maze cognition in middle-aged mice. **(A)** Latency to reach the platform. Blue asterisks refer to statistically significant differences between the T1 and T2 groups; red asterisks refer to differences between the T2 and T3 groups. **(B)** Swim path recorded by the video camera. **(C)** Velocity during the probe trial. There was no significant difference among the T1, T2, and T3 groups.** (D)** Number of times crossing the platform (left) and time spent in the target quadrant (right) were significantly increased in the T2 group compared with the T1 or T3 group. Data are presented as means ± standard error of the mean (SEM). *P < 0.05, **P < 0.01, and ***P < 0.001.

The results of the Morris water maze task during the five consecutive training days are presented in Figures 2A,B. Two-way ANOVA for repeated measures showed a significant interaction between the group factor and the day of training factor (*F* = 3.786, *P* < 0.0001). The main effects of the group factor (*F* = 10.60, *P* < 0.001) and the time factor (*F* = 42.28, *P* < 0.001) were also significant. On day 1, the latencies to reach the platform (mean ± SEM) in the T1/T2/T3 groups were (51.686 ± 0.959 s)/(50.324 ± 1.243 s)/(51.950 ± 0.972 s). There was no significant difference in latency to reach the platform among the three groups on day 1 (*n* = 12/group, *F*_(2,141)_ = 0.670, *P* = 0.513). However, there were significant differences on the second day (*F*_(2,141)_ = 22.986, *P* < 0.001), third day (*F*_(2,141)_ = 8.638, *P* < 0.001), fourth day (*F*_(2,141)_ = 26.207, *P* < 0.001) and fifth day (*F*_(2,141)_ = 59.213, *P* < 0.001). On day 2, the latencies to reach the platform in the T1/T2/T3 groups were (44.948 ± 1.460 s)/(34.562 ± 1.318 s)/(46.926 ± 1.374 s). Pairwise comparisons showed a significantly decreased latency to reach the platform in the T2 group compared with the T1 group (*t* = −5.302, *P* < 0.001) or T3 group (*t* = −6.311, *P* < 0.001). However, there was no significant difference between the T1 and T3 groups (*t* = −1.010, *P* = 0.314). On day 3, the latencies to reach the platform in the T1/T2/T3 groups were (44.540 ± 1.458 s)/(35.716 ± 1.713 s)/(38.566 ± 1.408 s), respectively. Pairwise comparisons showed that the latency significantly decreased both in the T2 (*t* = −4.072, *P* < 0.001) and T3 groups (*t* = −2.757, *P* = 0.007) compared with the T1 group, but there was no significant difference between the T2 and T3 groups (*t* = −1.315, *P* = 0.191). On day 4, the latencies to reach the platform in the T1/T2/T3 groups were (33.787 ± 1.456 s)/(18.622 ± 1.778 s)/(30.531 ± 1.419 s). The latency in the T2 group was significantly decreased compared with the T1 (*t* = −6.876, *P* < 0.001) and T3 group (*t* = −5.400, *P* < 0.001), but there was no significant difference between the T1 and T3 groups (*t* = 1.476, *P* = 0.142). On the last training day, the latencies to reach the platform in the T1/T2/T3 groups were (34.646 ± 1.348 s)/(16.222 ± 1.256 s)/(29.447 ± 1.085 s). Pairwise comparisons showed that the T2 group had a significant decrease in the latency to reach the platform compared with the T1 (*t* = −10.554, *P* < 0.001) or T3 group (*t* = −7.576, *P* < 0.001). Furthermore, the latency to reach the platform in the T3 group was significantly decreased compared with the T1 group (*t* = −2.978, *P* = 0.003). As seen in Figure 2C, there was no significant difference of the velocities among the T1 (3.385 ± 0.153 cm/s) T2 (3.428 ± 0.141 cm/s), and T3 (3.513 ± 0.241 cm/s) groups (*F*_(2,33)_ = 0.127, *P* = 0.882) in the probe trial. During the probe trial (Figure 2D), the number of times crossing the platform was significantly different among the three groups (*F*_(2,33)_ = 15.134, *P* < 0.001); it was increased in the T2 group (8.250 ± 0.494) compared with the T1 (4.250 ± 0.494; *t* = 5.092, *P* < 0.001) and T3 groups (4.833 ± 0.661; *t* = 4.350, *P* < 0.001). However, there was no significant difference between the T1 and T3 groups (*t* = −0.743, *P* = 0.463). Similarly, the time spent in the target quadrant was significantly different among the three groups (*F*_(2,33)_ = 7.234, *P* < 0.001); it was significantly increased in the T2 group (53.234 ± 4.120%) compared with the T1 (35.614 ± 2.668%; *t* = 3.795, *P* < 0.001) or T3 group (43.420 ± 2.870%; *t* = 2.114, *P* = 0.038), but there was no significant difference between the T1 and T3 groups (*t* = −1.681, *P* = 0.097).

### Neurogenesis and Gliogenesis in the Dentate Gyrus

**Figure 3:**
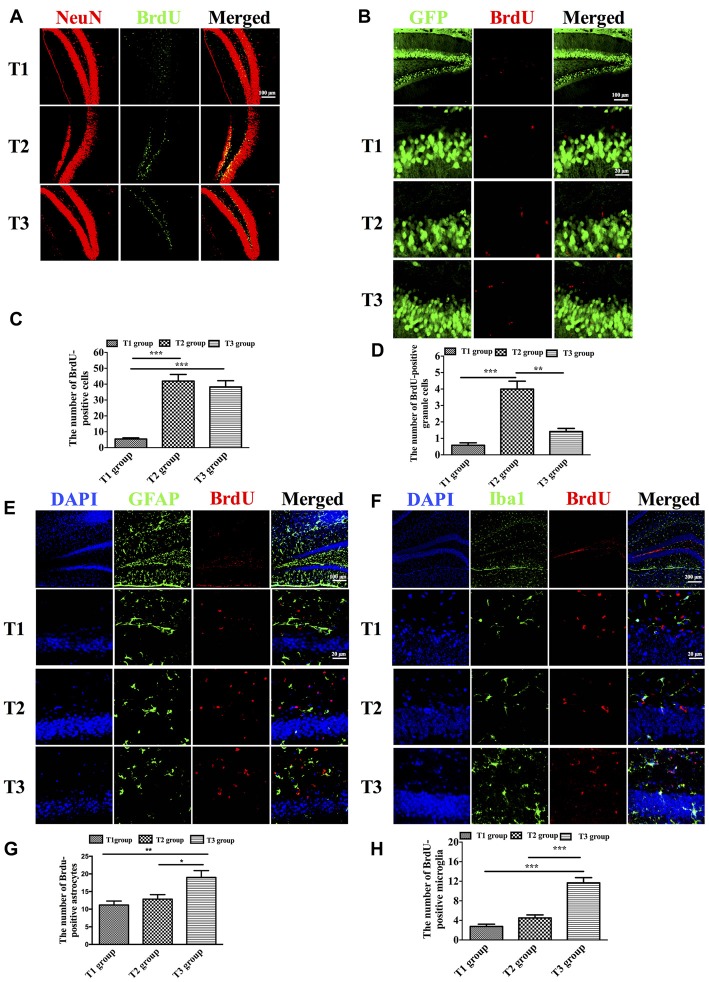
Intermittent voluntary wheel running facilitated newborn cells to differentiate into granule cells in the dentate gyrus (DG) in middle-aged mice. **(A)** Representative images of newborn neurons in the DG labeled by anti-BrdU and anti-NeuN. Scale bar = 100 μm.** (B)** The number of BrdU-positive cells was significantly increased in the T2 and T3 groups compared with the T1 group. **(C)** Representative images of newborn granule cells, which were labeled by anti-BrdU and green fluorescent protein (GFP) in Thy1-GFP transgenic mice. Low magnification: scale bar = 100 μm; high magnification: scale bar = 20 μm.** (D)** The BrdU-positive granule cells were significantly increased in the T2 group compared with the T1 and T3 groups.** (E)** Representative images of newborn astrocytes labeled by anti-BrdU and anti-glial fibrillary acidic protein (anti-GFAP). Low magnification: scale bar = 100 μm; high magnification: scale bar = 20 μm.** (F)** The BrdU-positive astrocytes were significantly increased in the T3 group compared with the T1 and T2 groups.** (G)** Representative images of repopulated microglia labeled by anti-BrdU and anti-Iba1. Low magnification: scale bar = 200 μm; high magnification: scale bar = 20 μm.** (H)** The number of BrdU-positive microglia was significantly increased in the T3 group compared with the T1 and T2 groups. Data are presented as means ± SEM. *P < 0.05, **P < 0.01, and ***P < 0.001.

We used anti-BrdU to label newborn cells in the DG. We observed that the number of BrdU-positive cells among the three groups was significantly different (Figures 3A,B; *F*_(2,33)_ = 35.834, *P* < 0.001). BrdU-positive cells were significantly increased in the T2 (41.917 ± 4.209; *t* = 7.687, *P* < 0.001) and T3 (38.250 ± 3.939; *t* = 6.915, *P* < 0.001) groups compared with the T1 group (5.416 ± 0.763), whereas there was no significant difference between the T2 and T3 groups (*t* = 0.772, *P* = 0.445). Next, we used Thy1-GFP transgenic mice to study newborn granule cells. Since Thy-1 is a promoter for neuronal expression, granule cells were specifically labeled with GFP in Thy1-GFP transgenic mice. The number of BrdU-positive granule cells was significantly different among the three groups (Figures 3C,D; *F*_(2,33)_ = 33.218, *P* < 0.001). There was a significant increase of BrdU-positive granule cells in the T2 group (4.000 ± 0.477) compared with the T1 (0.583 ± 0.149; *t* = 7.819, *P* < 0.001) and T3 groups (1.417 ± 0.193; *t* = 5.911, *P* < 0.001), whereas there was no significant difference between the T1 and T3 groups (*t* = −1.906, *P* = 0.065). Further, we study gliogenesis using anti-GFAP for astrocytes and anti-Iba1 for microglia. We observed that the number of BrdU-positive astrocytes among the three groups was significantly different (Figures 3E,F; *F*_(2,33)_ = 7.675, *P* = 0.005). There was a significant increase of BrdU-positive astrocytes in the T3 group (19.000 ± 1.932) compared with the T1 (11.17 ± 1.138; *t* = 3.719, *P* = 0.002) and T2 groups (12.83 ± 1.276; *t* = 2.928, *P* = 0.010), whereas there was no significant difference between the T1 and T2 groups (*t* = −0.792, *P* = 0.441). As to microgliogenesis, we found that the number of BrdU-positive microglia was significantly different among the three groups (Figures 3G,H; *F*_(2,33)_ = 37.468, *P* < 0.001). *Post hoc* tests revealed a significant increase in BrdU-positive microglia in the T3 group (11.667 ± 1.082) compared with the T1 (2.750 ± 0.479; *t* = 8.166, *P* < 0.001) or T2 (4.500 ± 0.622) group (*t* = 6.563, *P* < 0.001), but there was no significant difference between the T1 and T2 groups (*t* = −1.603, *P* = 0.118).

### Correlation Analysis

To assess the relationship between the newly-generated cells and performance in the water maze task, a Pearson’s correlation was performed (Table 1). There was a significant negative correlation between BrdU-positive cells and latency to the platform (*r* = −0.602, *P* < 0.001). There was no correlation between BrdU-positive cells and velocity during the probe trial. A significant positive correlation was found between BrdU-positive cells and number of times crossing the platform (*r* = 0.375, *P* = 0.024). A significant positive correlation was also found between BrdU-positive cells and time spent in target quadrant (*r* = 0.344, *P* = 0.040). There was a significant negative correlation between BrdU-positive granule cells and latency to the platform (*r* = −0.916, *P* < 0.001). Neither velocity during the probe trial nor time spent in target quadrant had a significant correlation with BrdU-positive granule cells. There was a significant positive correlation between BrdU-positive granule cells and number of times crossing the platform (*r* = 0.740, *P* < 0.001). However, as to gliogenesis, no correlation was found between performance in the water maze task and BrdU-positive astrocytes or microglia.

**Table 1:** Correlations between measures of newborn cells and performance in the Morris water maze task.

	BrdU^+^ cells	BrdU^+^ granule cells	BrdU^+^ astrocytes	BrdU^+^ microglia
Latency to the platform	*r* = −0.602, *p* < 0.001	*r* = −0.916, *p* < 0.001	n.s.	n.s.
Velocity during the probe trial	n.s.	n.s.	n.s.	n.s.
Number of times crossing the platform	*r* = 0.375, *p* = 0.024	*r* = 0.740, *p* < 0.001	n.s.	n.s.
Time spent in target quadrant	*r* = 0.344, *p* = 0.040	n.s.	n.s.	n.s.

*n.s., non-siginicant*.

## Discussion

In this study, we tested the effects of different voluntary wheel-running types on hippocampus-dependent cognition and hippocampal neurogenesis in middle-aged mice. Our results showed that intermittent voluntary wheel running was more beneficial for improving spatial memory than continuous voluntary wheel running. Moreover, we found that intermittent but not continuous voluntary wheel running could promote newborn cells to differentiate into granule cells. These results support the hypothesis that the improvement in spatial memory is associated with hippocampal neurogenesis induced by intermittent voluntary wheel running in middle-aged mice.

To evaluate spatial cognitive function, we performed the Morris water maze task. The results showed that intermittent, but not continuous voluntary wheel running, had more beneficial effects on water maze cognition in middle-aged mice. Similar results were observed following forced exercise. It was found that mild treadmill running training is more beneficial for improving spatial memory compared with intense treadmill running training (Inoue et al., 2015). It is now recognized that neural plasticity in the form of hippocampal neurogenesis occurs throughout adulthood, even in the elderly (Zhao et al., 2008), which is closely related to the maintenance and improvement of hippocampal-dependent cognitive function (Drapeau et al., 2003). Thus, we hypothesized that the beneficial effects of intermittent voluntary exercise on cognition may be caused by increased hippocampal neurogenesis. In our study, newborn cells were labeled with BrdU, a thymidine analog incorporated into the genetic material during the synthetic DNA phase (S phase) of mitotic division. We found that the number of BrdU-positive cells was significantly increased in both intermittent and continuous voluntary wheel-running groups compared with the no voluntary wheel-running group (Figure 3B), indicating that voluntary wheel running could enhance the survival of newborn cells and that the number of surviving newborn cells is not dependent on the duration of voluntary wheel running. These data are consistent with previous studies (Trejo et al., 2001; Motta-Teixeira et al., 2016).

Further, we studied another important component of hippocampal neurogenesis, the differentiation of newborn cells. Thy1-GFP transgenic mice were used to evaluate granule cells neurogenesis specifically. The results suggested that intermittent wheel running facilitated survival of newborn granule cells (Figures 3C,D), which could structurally and functionally integrate into the existing cellular networks (Toni and Sultan, 2011; Mongiat and Schinder, 2014) and contribute to hippocampus-dependent spatial cognition (Aimone et al., 2010; Radic et al., 2015). We speculated that the cognitive improvement in intermittently-exercised mice might be due to the increased neurogenesis. To prove our hypothesis, we performed correlation analysis to decipher the relationship between neurogenesis and behavior improvement. We found that neurogenesis had a significant correlation with performance in the water maze task (Table 1). Such correlation between hippocampal neurogenesis and spatial cognition has been observed in several studies. Mice with decreased hippocampal neurogenesis due to genetic regulation (Zhao et al., 2003) or aging (Drapeau et al., 2003; Driscoll et al., 2006) have impaired performance in the Morris water maze. In contrast, mice with increased neurogenesis due to voluntary running (van Praag et al., 1999) or enriched environment (Kempermann et al., 1997) have improved learning and memory function. In the present study, there were just a few BrdU-positive cells in high magnification (Figures 3C,D), but the results were consistent with previous studies that a low number of BrdU-positive neurons were observed (Kee et al., 2007b). Moreover, it has been confirmed that only a small fraction of neurons was used during the water maze task (Kee et al., 2007a). Meanwhile, new granule cells were more likely to be recruited into circuits supporting spatial memory than existing granule cells (Kee et al., 2007b; Clark et al., 2008). Therefore, this preferential recruitment supported our speculation that increased granule cells neurogenesis made a unique contribution to cognitive improvement in intermittently-exercised mice.

It has been reported that glial cells make up a larger proportion of newborn cells in aged mice compared with young mice (van Praag et al., 2005). We further studied gliogenesis, including astrogliogenesis and microgliogenesis. As to astrogliogenesis, our results demonstrated that continuous wheel running could increase newly-generated astrocytes (Figures 3E,F). Since in the adult subgranular zone, only the neuronal and astroglial lineages could be generated from neural stem cells (NSC; Suh et al., 2007; Bonaguidi et al., 2011), we hypothesized that the decrease in neuronal differentiation in the continuous voluntary running group was due to an increase in the number of new cells that differentiated into astrocytes. Given that the newborn neurons were positively correlated with spatial cognition, this explained why behavior performance in the continuous running group was not as good as that in the intermittent running group. In the current study, we also found that continuous wheel running facilitated repopulation of microglia (Figures 3G,H). It has been reported that repopulated microglia are derived from pre-existing microglia, rather than progenitor cells (Huang et al., 2018). As the resident immune cells in the brain, microglia mediate the inflammation-induced reduction in neurogenesis and are the key contributor to the acceleration of cognitive decline in aged mice (Gebara et al., 2013; Wu et al., 2016). The experimental activation of microglia has been shown to decrease adult neurogenesis by specifically inhibiting the proliferation or the survival of new cells and lead to NSC-derived astrogliogenesis (Monje et al., 2003; Cacci et al., 2005; Fujioka and Akema, 2010). Hence, in the current study, the availability of a larger number of new neurons, rather than glia, might facilitate improved spatial cognition in the intermittent wheel-running group.

Another possibility for the observed phenomenon is that the activation of progression from NSCs to lineage-committed progenitors to their progeny was different between intermittent and continuous running. It has been reported that the effects of short-term and long-term running in adult hippocampal neurogenesis could be different. Short-term (2 weeks) wheel-running paradigms promoted an increase of activated type 1 cells rather than an increase in number of cells, thus suggesting that running recruited quiescent NSCs rather than expanding the pool through symmetric division (Lugert et al., 2010; Gebara et al., 2016). However, long-term running failed to induce an increase of activated NSCs (Kronenberg et al., 2003; Steiner et al., 2008). Interestingly, it has been shown that at the end of short-term running, there was a decrease in the cell cycle length of type 2 and 3 cells (Farioli-Vecchioli et al., 2014). In our study, we speculated that intermittent voluntary running, namely repeated short-term running, might benefit from activated type 1 cells and a decrease in the cell cycle length. It will be of substantial interest in the future to investigate the cellular and molecular components related to neurogenesis and cognition.

## Author Contributions

Y-QH, CW, X-FH, DW, XH and ZP contributed to the conception and design of the study and interpretation of the data. Y-QH, CW, X-FH, DW, XH, F-YL and G-YD performed the research. Y-QH and X-FH conducted the statistical analyses. ZP provided additional statistical expertise related to the analysis. Y-QH and G-QX drafted the manuscript. YL and G-QX was the principal investigator of the study and was responsible for the study conception and interpretation of data and had final responsibility for the decision to submit for publication. All authors provided final approval for the version of the manuscript submitted for publication and agree to be accountable for the work.

## Conflict of Interest Statement

The authors declare that the research was conducted in the absence of any commercial or financial relationships that could be construed as a potential conflict of interest.

## References

[B1] Aimone J. B. Deng W. Gage F. H. (2010). Adult neurogenesis: integrating theories and separating functions. Trends Cogn. Sci. 14, 325–337. 10.1016/j.tics.2010.04.003 20471301 PMC2904863

[B2] Akers K. G. Martinez-Canabal A. Restivo L. Yiu A. P. De Cristofaro A. Hsiang H. L.. (2014). Hippocampal neurogenesis regulates forgetting during adulthood and infancy. Science 344, 598–602. 10.1126/science.1248903 24812394

[B3] Bonaguidi M. A. Wheeler M. A. Shapiro J. S. Stadel R. P. Sun G. J. Ming G.. (2011). *In vivo* clonal analysis reveals self-renewing and multipotent adult neural stem cell characteristics. Cell 145, 1142–1155. 10.1016/j.cell.2011.05.024 21664664 PMC3124562

[B4] Cacci E. Claasen J.-H. Kokaia Z. (2005). Microglia-derived tumor necrosis factor-α exaggerates death of newborn hippocampal progenitor cells *in vitro*. J. Neurosci. Res. 80, 789–797. 10.1002/jnr.20531 15884015

[B5] Canas P. M. Duarte J. M. Rodrigues R. J. Köfalvi A. Cunha R. A. (2009). Modification upon aging of the density of presynaptic modulation systems in the hippocampus. Neurobiol. Aging 30, 1877–1884. 10.1016/j.neurobiolaging.2008.01.003 18304697

[B6] Clark P. J. Brzezinska W. J. Thomas M. W. Ryzhenko N. A. Toshkov S. A. Rhodes J. S. (2008). Intact neurogenesis is required for benefits of exercise on spatial memory but not motor performance or contextual fear conditioning in C57BL/6J mice. Int. J. Mol. Sci. 155, 1048–1058. 10.1016/j.neuroscience.2008.06.051 18664375

[B7] Cotman C. W. Berchtold N. C. Christie L. A. (2007). Exercise builds brain health: key roles of growth factor cascades and inflammation. Trends Neurosci. 30, 464–472. 10.1016/j.tins.2007.06.011 17765329

[B8] Drapeau E. Mayo W. Aurousseau C. Le Moal M. Piazza P. V. Abrous D. N. (2003). Spatial memory performances of aged rats in the water maze predict levels of hippocampal neurogenesis. Proc. Natl. Acad. Sci. U S A 100, 14385–14390. 10.1073/pnas.2334169100 14614143 PMC283601

[B9] Driscoll I. Howard S. R. Stone J. C. Monfils M. H. Tomanek B. Brooks W. M.. (2006). The aging hippocampus: a multi-level analysis in the rat. Neuroscience 139, 1173–1185. 10.1016/j.neuroscience.2006.01.040 16564634

[B11] Farioli-Vecchioli S. Mattera A. Micheli L. Ceccarelli M. Leonardi L. Saraulli D.. (2014). Running rescues defective adult neurogenesis by shortening the length of the cell cycle of neural stem and progenitor cells. Stem Cells 32, 1968–1982. 10.1002/stem.1679 24604711

[B12] Foster T. C. (2006). Biological markers of age-related memory deficits: treatment of senescent physiology. CNS Drugs 20, 153–166. 10.2165/00023210-200620020-00006 16478290

[B13] Foster T. C. Kyritsopoulos C. Kumar A. (2017). Central role for NMDA receptors in redox mediated impairment of synaptic function during aging and Alzheimer’s disease. Behav. Brain Res. 322, 223–232. 10.1016/j.bbr.2016.05.012 27180169

[B14] Fujioka H. Akema T. (2010). Lipopolysaccharide acutely inhibits proliferation of neural precursor cells in the dentate gyrus in adult rats. Brain Res. 1352, 35–42. 10.1016/j.brainres.2010.07.032 20647006

[B15] Gebara E. Bonaguidi M. A. Beckervordersandforth R. Sultan S. Udry F. Gijs P.-J.. (2016). Heterogeneity of radial glia-like cells in the adult hippocampus. Stem Cells 34, 997–1010. 10.1002/stem.2266 26729510 PMC5340291

[B16] Gebara E. Sultan S. Kocher-Braissant J. Toni N. (2013). Adult hippocampal neurogenesis inversely correlates with microglia in conditions of voluntary running and aging. Front. Neurosci. 7:145. 10.3389/fnins.2013.00145 23970848 PMC3747329

[B17] Hillman C. H. Erickson K. I. Kramer A. F. (2008). Be smart, exercise your heart: exercise effects on brain and cognition. Nat. Rev. Neurosci. 9, 58–65. 10.1038/nrn2298 18094706

[B19] Huang Y. Xu Z. Xiong S. Sun F. Qin G. Hu G.. (2018). Repopulated microglia are solely derived from the proliferation of residual microglia after acute depletion. Nat. Neurosci. 21, 530–540. 10.1038/s41593-018-0090-8 29472620

[B20] Hullinger R. Puglielli L. (2017). Molecular and cellular aspects of age-related cognitive decline and Alzheimer’s disease. Behav. Brain Res. 322, 191–205. 10.1016/j.bbr.2016.05.008 27163751 PMC5099115

[B21] Inoue K. Okamoto M. Shibato J. Lee M. C. Matsui T. Rakwal R.. (2015). Long-term mild, rather than intense, exercise enhances adult hippocampal neurogenesis and greatly changes the transcriptomic profile of the hippocampus. PLoS One 10:e0128720. 10.1371/journal.pone.0128720 26061528 PMC4464753

[B22] Kee N. Teixeira C. M. Wang A. H. Frankland P. W. (2007a). Imaging activation of adult-generated granule cells in spatial memory. Nat. Protoc. 2, 3033–3044. 10.1038/nprot.2007.415 18079702

[B23] Kee N. Teixeira C. M. Wang A. H. Frankland P. W. (2007b). Preferential incorporation of adult-generated granule cells into spatial memory networks in the dentate gyrus. Nat. Neurosci. 10, 355–362. 10.1038/nn1847 17277773

[B24] Kempermann G. Kuhn H. G. Gage F. H. (1997). More hippocampal neurons in adult mice living in an enriched environment. Nature 386, 493–495. 10.1038/386493a0 9087407

[B25] Koike H. Ibi D. Mizoguchi H. Nagai T. Nitta A. Takuma K.. (2009). Behavioral abnormality and pharmacologic response in social isolation-reared mice. Behav. Brain Res. 202, 114–121. 10.1016/j.bbr.2009.03.028 19447287

[B26] Kronenberg G. Reuter K. Steiner B. Brandt M. D. Jessberger S. Yamaguchi M.. (2003). Subpopulations of proliferating cells of the adult hippocampus respond differently to physiologic neurogenic stimuli. J. Comp. Neurol. 467, 455–463. 10.1002/cne.10945 14624480

[B27] Lichtenwalner R. J. Forbes M. E. Bennett S. A. Lynch C. D. Sonntag W. E. Riddle D. R. (2001). Intracerebroventricular infusion of insulin-like growth factor-I ameliorates the age-related decline in hippocampal neurogenesis. Neuroscience 107, 603–613. 10.1016/s0306-4522(01)00378-5 11720784

[B28] Lugert S. Basak O. Knuckles P. Haussler U. Fabel K. Götz M.. (2010). Quiescent and active hippocampal neural stem cells with distinct morphologies respond selectively to physiological and pathological stimuli and aging. Cell Stem Cell 6, 445–456. 10.1016/j.stem.2010.03.017 20452319

[B29] Luo C. X. Jiang J. Zhou Q. G. Zhu X. J. Wang W. Zhang Z. J.. (2007). Voluntary exercise-induced neurogenesis in the postischemic dentate gyrus is associated with spatial memory recovery from stroke. J. Neurosci. Res. 85, 1637–1646. 10.1002/jnr.21317 17465031

[B30] Mongiat L. A. Schinder A. F. (2014). Neuroscience. A price to pay for adult neurogenesis. Science 344, 594–595. 10.1126/science.1254236 24812393

[B31] Monje M. L. Toda H. Palmer T. D. (2003). Inflammatory blockade restores adult hippocampal neurogenesis. Science 302, 1760–1765. 10.1126/science.1088417 14615545

[B32] Motta-Teixeira L. C. Takada S. H. Machado-Nils A. V. Nogueira M. I. Xavier G. F. (2016). Spatial learning and neurogenesis: effects of cessation of wheel running and survival of novel neurons by engagement in cognitive tasks. Hippocampus 26, 794–803. 10.1002/hipo.22560 26669934

[B33] Nishijima T. Llorens-Martín M. Tejeda G. S. Inoue K. Yamamura Y. Soya H.. (2013). Cessation of voluntary wheel running increases anxiety-like behavior and impairs adult hippocampal neurogenesis in mice. Behav. Brain Res. 245, 34–41. 10.1016/j.bbr.2013.02.009 23428744

[B34] Radic T. Al-Qaisi O. Jungenitz T. Beining M. Schwarzacher S. W. (2015). Differential structural development of adult-born septal hippocampal granule cells in the thy1-gfp mouse, nuclear size as a new index of maturation. PLoS One 10:e0135493. 10.1371/journal.pone.0135493 26267362 PMC4534292

[B35] Rhodes J. S. van Praag H. Jeffrey S. Girard I. Mitchell G. S. Garland T. Jr.. (2003). Exercise increases hippocampal neurogenesis to high levels but does not improve spatial learning in mice bred for increased voluntary wheel running. Behav. Neurosci. 117, 1006–1016. 10.1037/0735-7044.117.5.1006 14570550

[B36] Shen X. Li A. Zhang Y. Dong X. Shan T. Wu Y.. (2013). The effect of different intensities of treadmill exercise on cognitive function deficit following a severe controlled cortical impact in rats. Int. J. Mol. Sci. 14, 21598–21612. 10.3390/ijms141121598 24185909 PMC3856023

[B37] Shih P. C. Yang Y. R. Wang R. Y. (2013). Effects of exercise intensity on spatial memory performance and hippocampal synaptic plasticity in transient brain ischemic rats. PLoS One 8:e78163. 10.1371/journal.pone.0078163 24205142 PMC3808358

[B38] Shimada H. Hamakawa M. Ishida A. Tamakoshi K. Nakashima H. Ishida K. (2013). Low-speed treadmill running exercise improves memory function after transient middle cerebral artery occlusion in rats. Behav. Brain Res. 243, 21–27. 10.1016/j.bbr.2012.12.018 23266325

[B39] Small S. A. Tsai W. Y. DeLaPaz R. Mayeux R. Stern Y. (2002). Imaging hippocampal function across the human life span: is memory decline normal or not? Ann. Neurol. 51, 290–295. 10.1002/ana.10105 11891823

[B41] Steiner B. Zurborg S. Hörster H. Fabel K. Kempermann G. (2008). Differential 24 h responsiveness of Prox1-expressing precursor cells in adult hippocampal neurogenesis to physical activity, environmental enrichment and kainic acid-induced seizures. Neuroscience 154, 521–529. 10.1016/j.neuroscience.2008.04.023 18502050

[B42] Stranahan A. M. Khalil D. Gould E. (2006). Social isolation delays the positive effects of running on adult neurogenesis. Nat. Neurosci. 9, 526–533. 10.1038/nn1668 16531997 PMC3029943

[B43] Suh H. Consiglio A. Ray J. Sawai T. D’Amour K. A. Gage F. H. (2007). *In vivo* fate analysis reveals the multipotent and self-renewal capacities of sox2^+^ neural stem cells in the adult hippocampus. Cell Stem Cell 1, 515–528. 10.1016/j.stem.2007.09.002 18371391 PMC2185820

[B44] Toni N. Sultan S. (2011). Synapse formation on adult-born hippocampal neurons. Eur. J. Neurosci. 33, 1062–1068. 10.1111/j.1460-9568.2011.07604.x 21395849

[B45] Trejo J. L. Carro E. Torres-Aleman I. (2001). Circulating insulin-like growth factor I mediates exercise-induced increases in the number of new neurons in the adult hippocampus. J. Neurosci. 21, 1628–1634. 10.1523/JNEUROSCI.21-05-01628.2001 11222653 PMC6762955

[B46] van Praag H. Kempermann G. Gage F. H. (1999). Running increases cell proliferation and neurogenesis in the adult mouse dentate gyrus. Nat. Neurosci. 2, 266–270. 10.1038/6368 10195220

[B47] van Praag H. Shubert T. Zhao C. Gage F. H. (2005). Exercise enhances learning and hippocampal neurogenesis in aged mice. J. Neurosci. 25, 8680–8685. 10.1523/JNEUROSCI.1731-05.2005 16177036 PMC1360197

[B48] Wu Z. Yu J. Zhu A. Nakanishi H. (2016). Nutrients, microglia aging, and brain aging. Oxid. Med. Cell. Longev. 2016:7498528. 10.1155/2016/7498528 26941889 PMC4752989

[B49] Zhao C. Deng W. Gage F. H. (2008). Mechanisms and functional implications of adult neurogenesis. Cell 132, 645–660. 10.1016/j.cell.2008.01.033 18295581

[B50] Zhao X. Ueba T. Christie B. R. Barkho B. McConnell M. J. Nakashima K.. (2003). Mice lacking methyl-CpG binding protein 1 have deficits in adult neurogenesis and hippocampal function. Proc. Natl. Acad. Sci. U S A 100, 6777–6782. 10.1073/pnas.1131928100 12748381 PMC164523

